# Prognostic Value of the Geriatric Nutritional Risk Index and Controlling Nutritional Status Score in Patients Aged ≥ 75 Years With Heart Failure With Preserved Ejection Fraction

**DOI:** 10.14740/cr2282

**Published:** 2026-10-20

**Authors:** Yao Li, Hai Rui Shao, Ying Liu, Jing Yu Wang, Yu Gang Yin, Chun Yang, Xian Wang, Yong Zhong, Lei Lv

**Affiliations:** a Department of Geriatric Cardiology, Affiliated Jinling Hospital, Medical School of Nanjing University, Nanjing, Jiangsu 210002, China; b Department of Health Medicine, Affiliated Jinling Hospital, Medical School of Nanjing University, Nanjing, Jiangsu 210002, China

**Keywords:** Malnutrition, Geriatric Nutritional Risk Index, CONUT score, Elderly, Heart failure with preserved ejection fraction, Prognosis

## Abstract

**Background:**

Malnutrition is common in elderly patients with heart failure with preserved ejection fraction (HFpEF), but its prognostic significance and the optimal screening tool remain unclear. This retrospective, single-center study aimed to compare the application of the Geriatric Nutritional Risk Index (GNRI) and the Controlling Nutritional Status (CONUT) score in assessing nutritional status in very elderly patients with HFpEF, and to evaluate the association between nutritional status and clinical outcomes.

**Methods:**

A total of 196 patients aged ≥ 75 years (mean age 92.12 ± 4.28 years, 86.2% male) with HFpEF were enrolled. Nutritional status was categorized using CONUT and GNRI according to standard cut-offs.

**Results:**

Patients with higher CONUT scores or lower GNRI scores (indicating worse nutritional status) had significantly longer hospital stays, lower levels of nutrition-related indices (albumin, total cholesterol, low-density lipoprotein cholesterol (LDL-C), serum ferritin, transferrin saturation), lower lymphocyte counts, and higher NT-pro-B-type natriuretic peptide (NT-proBNP) and inflammatory markers (C-reactive protein (CRP), procalcitonin (PCT), interleukin-6 (IL-6)) (all P < 0.05). Over a median follow-up of 11.5 months, 83 patients (42.3%) died. In unadjusted analyses, patients with worse nutritional status had significantly higher rates of all-cause death, all-cause readmission, and heart failure-related readmission (all P < 0.05). Multivariable Cox regression revealed that CONUT (hazard ratio (HR) = 1.366, 95% confidence interval (CI): 1.181–1.579, P < 0.001) was an independent risk factor for all-cause mortality, while GNRI (HR = 0.962, 95% CI: 0.927–0.999, P = 0.043) was a protective factor. The CONUT and GNRI showed poor consistency (κ = 0.235, P < 0.01). Using receiver operating characteristic (ROC) analysis based on vital status at the end of follow-up, the area under the curve (AUC) for predicting all-cause mortality was 0.86 (95% CI: 0.80–0.91) for CONUT, 0.79 (95% CI: 0.73–0.86) for GNRI, and 0.87 (95% CI: 0.82–0.92) for a combined model incorporating both scores via logistic regression. Net reclassification improvement (NRI) analysis showed that compared with the GNRI score, the NRI of the CONUT score was 0.580 (95% CI: 0.309–0.852), and the NRI of the combined score was 0.760 (95% CI: 0.499–1.022), both reaching statistical significance (P < 0.01). By contrast, the NRI was 0.083 (95% CI: −0.197 to 0.363, P = 0.560) for the combined score versus the CONUT score.

**Conclusions:**

Malnutrition is common in elderly patients with HFpEF. CONUT and GNRI scores are associated with adverse outcomes. Compared with GNRI alone, the combined assessment of the two scores improves predictive performance for adverse prognosis in elderly patients with HFpEF, while no incremental benefit is observed over CONUT alone.

## Introduction

Heart failure (HF) is the severe and terminal stage of various heart diseases. Elderly HF patients usually have a poorer prognosis due to comorbidities and physiological decline [1]. Heart failure with preserved ejection fraction (HFpEF) is the most prevalent phenotype among elderly patients, characterized by left ventricular ejection fraction ≥ 50%, typical signs and/or symptoms of HF, and objective evidence [2]. Nevertheless, improving their prognosis remains a critical challenge in geriatric cardiology.

Malnutrition is common in elderly HFpEF patients and arises from metabolic disorders, gastrointestinal dysfunction, and underlying diseases. Nutritional assessment is emphasized in various diseases and has been proven to be associated with disease prognosis. The Controlling Nutritional Status (CONUT) and Geriatric Nutritional Risk Index (GNRI) scores are common tools for evaluating nutritional status [3], with the former derived from serum albumin (ALB), total cholesterol (TC), and lymphocyte count (LY) [4], and the latter from body mass index (BMI) and serum ALB [5]. Recent studies have demonstrated that GNRI, CONUT, and related nutritional scores are significantly associated with adverse outcomes in elderly patients with acute ischemic stroke [6], geriatric orthopedic trauma requiring intensive care [7], and hip fracture patients undergoing surgery [8]. Current evidence also indicates that malnutrition is linked to poor prognosis in elderly HF patients [9, 10], yet most evidence comes from general HF or heart failure with reduced ejection fraction (HFrEF) populations. Therefore, we hypothesized that both CONUT and GNRI would be significantly associated with adverse prognosis in HFpEF patients aged ≥ 75 years, and that a combined assessment of the two scores might provide incremental predictive value over either index alone. This study aimed to compare GNRI and CONUT scores for malnutrition assessment in HFpEF patients aged ≥ 75 years, and investigate the association between malnutrition and adverse prognosis, so as to identify suitable nutritional assessment tools.

## Materials and Methods

### Study population

This was a single-center, retrospective observational study conducted in the Geriatric Ward of Jinling Hospital. Consecutive patients aged ≥ 75 admitted to the Geriatric Ward of Jinling Hospital for cardiovascular diseases from November 2021 to June 2024 were enrolled. All participants were uniformly diagnosed with HFpEF in accordance with the 2023 ESC Guidelines [11]. The diagnosis required: (1) typical signs and/or symptoms of HF; (2) LVEF ≥ 50%; (3) objective evidence, as indicated by elevated natriuretic peptides or echocardiographic evidence of structural or functional cardiac abnormalities. Since patient enrollment began before the guidelines were published, all cases enrolled earlier were retrospectively re-adjudicated using the same 2023 ESC criteria to maintain uniform diagnostic standards throughout the study. For patients with multiple admissions during the study period, only the first hospitalization was included as the index admission. To ensure the reliability of research data, all patients were required to complete a nutritional status assessment within 24 h of admission, with complete clinical data and follow-up information available. Exclusion criteria were as follows: (1)malignant tumors, hematological diseases, end-stage hepatic or renal insufficiency, or severe infections; (2) cases with severe and non-recoverable missing core clinical data or complete loss to follow-up. The handling of missing data was standardized to eliminate internal inconsistency. Minor missing values in key variables (including nutritional assessment indices, laboratory parameters, and baseline clinical characteristics) were processed via multiple imputation by chained equations (MICE) under the missing-at-random (MAR) assumption. A total of five imputed datasets were generated, and the imputation model incorporated age, sex, comorbidities, laboratory indicators, echocardiographic parameters, and nutritional scores. In this cohort, missing data mainly occurred in individual biochemical and nutritional variables. Cases with a missing rate exceeding 20% for any critical variable were directly excluded to guarantee the validity of statistical analyses. No patients were excluded due to general incomplete information. A total of 10 patients were excluded from the final analysis because of critical missing data that could not be recovered or because they were completely lost to follow-up, and all exclusions were strictly based on the pre-defined missing data threshold.

### Nutritional status assessment and clinical data collection

The general information and clinical data of the patients upon admission were documented. The former encompassed gender, age, BMI, comorbidities, and baseline medications. The latter included cardiac Doppler ultrasound findings and early morning fasting blood tests within 24 h of admission such as LY, hemoglobin (HGB), creatinine (CRE), ALB, prealbumin (PA), triglycerides (TG), high-density lipoprotein cholesterol (HDL-C), low-density lipoprotein cholesterol (LDL-C), NT-pro-B-type natriuretic peptide (NT-proBNP) and C-reactive protein (CRP).

The nutritional status of enrolled patients was assessed by CONUT score and GNRI. Patients were categorized into four groups based on their CONUT scores which ranged from 0 to 12 points [4]: normal (0–1), mild (2–4), moderate (5–8), and severe malnutrition (9–12). GNRI [5] was calculated from BMI and ALB as follows: GNRI = 1.489 × ALB (g/L) + 41.7 × actual body mass (kg)/ideal body mass (kg). Ideal body mass was calculated based on the Lorentz equation: Male: 0.75 × height (cm) − 62.5; Female: 0.6 × height (cm) − 40. If actual body mass was greater than ideal body mass, the ratio between them was defined as 1. Patients were divided into four groups: no risk of malnutrition (GNRI > 98), low (92 ≤ GNRI ≤ 98), medium (82 ≤ GNRI < 92), and high risk (GNRI < 82). Higher CONUT scores or lower GNRI scores indicated worse nutritional status. Baseline clinical characteristics of patients with different nutritional status were compared. The consistency and differences of the two tools for assessing malnutrition were analyzed.

### Follow-up and endpoint events

Patients were followed up by telephone or outpatient visits, and the rates of all-cause death, cardiovascular death, all-cause readmission, and HF-related readmission were recorded. All-cause death was defined as death from any cause during the follow-up period. Cardiovascular death was defined as death directly attributable to cardiovascular causes, including death from worsening HF, acute myocardial infarction, fatal arrhythmias, or sudden cardiac death. All-cause readmission was defined as any unplanned hospitalization during the follow-up period. HF-related readmission was defined as an unplanned hospitalization with HF as the primary diagnosis. All deaths were verified through review of hospital medical records, death certificates, or contact with family members. Outcome events were adjudicated by two independent clinicians who were blinded to the patients’ nutritional status; any disagreements were resolved by consensus with a third senior cardiologist. For patients with multiple readmissions during the follow-up period, only the first HF-related readmission or the first all-cause readmission was counted as an event to avoid bias from correlated observations. Time to first event was used in the survival analysis. All-cause death was the primary outcome indicator in this study; patients who did not reach the primary endpoint were followed up until September 2024. The prognosis of elderly patients with HFpEF under different nutritional status was compared.

### Ethical considerations

The study’s protocol was approved by the Ethics Committee (2023DZKY-051-01) of Affiliated Jinling Hospital, Medical School of Nanjing University, and patient enrollment was carried out according to the principles of the Declaration of Helsinki. Informed consent to participate was obtained from all of the participants in the study.

### Statistical analysis

Normally distributed continuous variables were expressed as mean ± standard deviation, and intergroup comparisons were conducted via one-way analysis of variance (ANOVA). Non-normally distributed continuous variables were presented as median (interquartile range) (M (P25, P75)), with intergroup comparisons via Kruskal–Wallis test. Categorical variables were expressed as numbers (percentages) and were compared using Fisher’s exact test. Follow-up duration was reported as median with interquartile range (IQR) due to the skewed distribution. Kaplan–Meier survival analysis was used to evaluate the effect of nutritional status on the endpoint. Survival curves were compared using the two-sided log-rank test. To further assess the independent prognostic value of nutritional scores, multivariable Cox proportional hazards regression was performed to estimate the risk of all-cause mortality associated with CONUT and GNRI. Both scores were entered into the model as continuous variables. Hazard ratios (HRs) with 95% confidence intervals (CIs) were reported for all variables in the final model. The proportional-hazards assumption was tested using Schoenfeld residuals. Agreement between diagnoses of the two tools for assessing malnutrition was assessed using the κ coefficient. The diagnostic performance of each nutritional indicator for clinical outcomes was assessed by receiver operating characteristic (ROC) curves, and the differences in diagnostic performance were compared using the area under the curve (AUC); the results were further validated using net reclassification improvement (NRI) analysis. Predicted probabilities for each model were generated using binary logistic regression. Statistical analyses were performed with the software SPSS 26.0. P values < 0.05 were considered statistically significant.

## Results

### Characteristics of patients

A total of 196 patients over 75 years with HFpEF were enrolled, including 182 males and 14 females, with a mean age of 92.12 ± 4.28 years. The patient flowchart is as shown in Figure 1.

**Figure 1:**
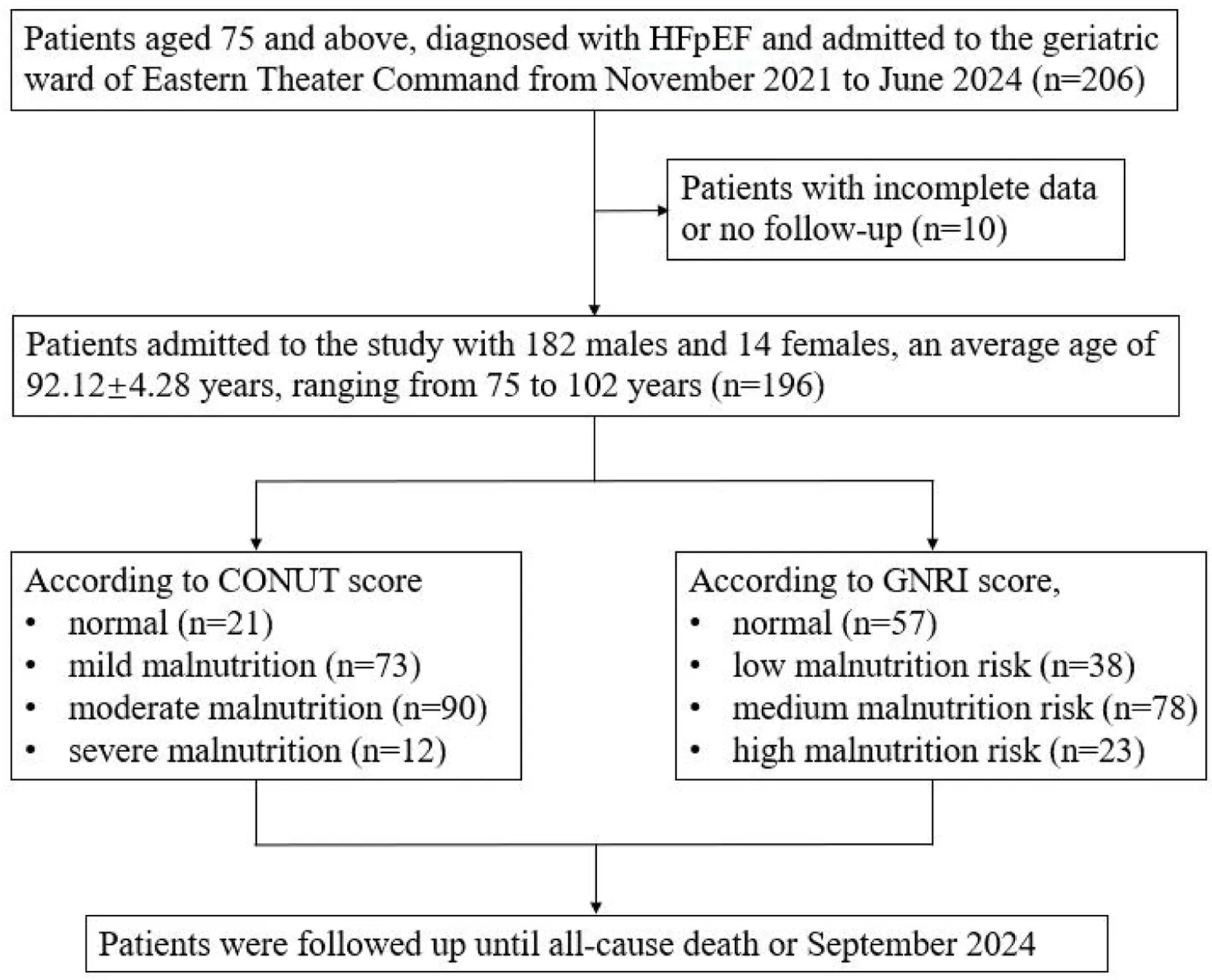
Patient flowchart. HFpEF: heart failure with preserved ejection fraction; CONUT: Controlling Nutritional Status; GNRI: Geriatric Nutritional Risk Index.

### Clinical data of different groups of patients under CONUT score and GNRI score

Patients in the higher CONUT group had longer hospital stays, higher proportions of patients with anemia, and receiving nutritional support therapy. Nutrition-related indicators (e.g., ALB, PA, HGB, total cholesterol, LDL-C, serum ferritin (SF), transferrin saturation (TSAT)), and immune indicators (lymphocytes) were significantly lower, while inflammatory indicators (CRP, PCT, IL-6) and cardiac function indicators (NT-proBNP) were significantly higher (all P < 0.05). No significant differences were observed in echocardiographic parameters among CONUT groups. A similar trend in clinical characteristics was observed with decreasing GNRI scores (higher nutritional risk), consistent with CONUT groups. However, distinct differences included smaller left atrial diameter (LAD) and left ventricular diastolic diameter (LVDD) in the high nutritional risk group (P < 0.05), as well as significant variations in HDL-C, interventricular septum (IVS), and left ventricular posterior wall (LVPW) across GNRI groups (P < 0.05), as shown in Table 1.

**Table 1:** Clinical Data of Patients Stratified by CONUT Score and GNRI Score

Characteristic	CONUT score				P value
	Normal (0–1, n = 21)	Mild malnutrition (2–4, n = 73)	Moderate malnutrition (5–8, n = 90)	Severe malnutrition (9–12, n = 12)	
General situation					
Hospitalization days (days)	18 (10, 33)	29 (17, 41)	31 (20, 47)^a^	46 (22, 55)^a^	0.011
Age (years)	93 (91, 95)	92 (90, 94)	92 (91, 94)	94 (92, 96)	0.318
Male (%)	20 (95.2)	67 (91.8)	85 (94.4)	10 (83.3)	0.413
Heart rate (beats/min)	70 (66, 78)	72 (66, 81)	73 (65, 80)	78 (75, 89)	0.078
Systolic blood pressure (mm Hg)	132 (120, 158)	132 (120, 144)	132 (120, 145)	123 (115, 145)	0.560
Diastolic blood pressure (mm Hg)	71 (62, 80)	71 (61, 78)	68 (62, 76)	73 (63, 77)	0.452
BMI (kg/m^2^)	22.0 (21.5, 24.6)	23.0 (22.5, 25.9)	22.4 (20.8, 24.2)	24.5 (22.0, 24.7)	0.289
CONUT score	1 (0, 1)	3 (2.5, 4)^a^	6 (5, 7)^a, b^	9 (9, 9)^a, b, c^	< 0.001
GNRI score	98.4 (96.1, 102.6)	96.5 (91.6, 105.0)^a^	88.0 (83.9, 91.4)^a, b^	82.1 (76.5, 87.1)^a, b, c^	< 0.001
Laboratory data					
Albumin (g/L)	38.4 ± 1.8	36.2 ± 4.0^a^	31.0 ± 3.0^a, b^	26.5 ± 2.6^a, b, c^	< 0.001
Lymphocyte (× 10^9^/L)	1.8 (1.7, 2.1)	1.5 (1.2, 1.9)^a^	1.0 (0.7, 1.2)^a, b^	0.7 (0.6, 1.4)^a, b^	< 0.001
Cholesterol (mmol/L)	4.6 (3.9, 5.5)	3.7 (2.9, 4.0)^a^	3.3 (2.9, 3.8)^a^	2.6 (2.5, 3.3)^a, b, c^	< 0.001
Hemoglobin (g/L)	124 (116, 131)	120 (100.5, 130)	107 (96.5, 120)^a, b^	99 (85.3, 121)^a, b^	< 0.001
C-reactive protein (mg/L)	0.5 (0.5, 3.5)	2.2 (0.5, 15.1)	14.5 (3.7, 52.2)^a, b^	13.6 (3.3, 50.6)^a, b^	< 0.001
Urea nitrogen (mmol/L)	8 (6.7, 12.5)	10.1 (7.6, 14)	9.2 (7.1, 13.6)	10.0 (6.7, 14)	0.419
Creatinine (µmol/L)	101.6 (80.2, 124.5)	110.4 (80.8, 174.5)	102.2 (79.6, 141.1)	100.8 (63.8, 135.4)	0.535
Prealbumin (mg/L)	229.2 ± 43.6	209.3 ± 51.6	185.3 ± 58.5^a, b^	150.5 ± 80.1^a, b^	<0.001
Triglyceride (mmol/L)	1.1 (0.9, 1.3)	1.0 (0.7, 1.3)	0.9 (0.6, 1.3)	0.7 (0.6, 1.1)	0.173
LDL-C (mmol/L)	2.4 (2.0, 3.0)	1.7 (1.3, 2.1)^a^	1.6 (1.3, 2.0)^a^	1.4 (1.1, 1.7)^a, b, c^	< 0.001
HDL-C (mmol/L)	1.2 (1.0, 1.5)	1.0 (0.8, 1.2)^a^	0.9 (0.8, 1.2)^a^	0.9 (0.8, 1.1)^a^	0.003
PCT (µg/L)	0.05 (0.03, 0.11)	0.07 (0.04, 0.11)	0.11 (0.06, 0.22)^a, b^	0.10 (0.06, 0.27)^a^	< 0.001
IL-6 (ng/L)	7.5 (3.7, 12.3)	10.7 (7.5, 20.9)^a^	18.9 (8.9, 37.8)^a, b^	36.6 (13.3, 56.7)^a, b^	< 0.001
NT-proBNP (pmol/L)	230.8 (143.8, 341.5)	269.5 (195, 392.9)	329.0 (225.5, 519.3)^a, b^	398.4 (242.8, 600.8)^a^	0.012
TSAT (%)	0.31 (0.24, 0.40)	0.26 (0.19, 0.34)	0.24 (0.18, 0.33)^a^	0.20 (0.15, 0.27)^a^	0.041
SF (µmol/L)	13.2 (11.6, 18.8)	11.6 (8.0, 13.8)^a^	9.1 (6.5, 11.5)^a, b^	8.1 (5.4, 10.3)^a, b^	< 0.001
UIBC (µmol/L)	31.8 (27.5, 38.1)	30.4 (23.5, 37.5)	25.5 (20.6, 32.1)^a, b^	28.4 (22.5, 32.9)	0.009
TIBC (µmol/L)	48.7 (42.9, 52.3)	42.9 (34.2, 51.1)^a^	35.7 (30.3, 41.4)^a, b^	36.1 (30.8, 41.9)^a^	< 0.001
Echocardiographic data					
LVEF (%)	63 (58.5, 69)	60 (57, 64)	61 (56, 66.3)	59.5 (55.8, 64)	0.180
AOD (mm)	34 (32, 37)	34 (32, 37.5)	35 (32.8, 38)	34.5 (33.3, 36.8)	0.797
LAD (mm)	43 (37.5, 45)	44 (38.5, 48.5)	41 (37, 45)	41 (39.3, 44.8)	0.072
LVDD (mm)	47 (42.5, 49.5)	47 (43.5, 50.5)	47 (44, 51)	45 (42.3, 46.8)	0.167
RVD (mm)	24 (21, 26)	25 (23, 26)	25 (22, 26)	24 (22.3, 26.8)	0.417
IVS (mm)	11 (10, 12)	11 (10, 12)	11 (10, 12)	11 (11, 13)	0.155
LVPW (mm)	10 (9, 11)	10 (10, 11)	10 (10, 11)	11 (10, 13)	0.284
Comorbidity					
Hypertension (%)	15 (71.4)	59 (80.8)	70 (77.8)	9 (75)	0.776
Diabetes (%)	5 (23.8)	32 (43.8)	40 (44.4)	5 (41.7)	0.373
Coronary heart disease (%)	20 (95.2)	70 (95.9)	85 (94.4)	12 (100)	0.940
COPD (%)	2 (9.5)	29 (39.7)^a^	31 (34.3)^a^	5 (41.7)^a^	0.055
Chronic renal insufficiency (%)	8 (38.1)	33 (45.2)	41 (45.6)	5 (41.7)	0.931
Anemia (%)	2 (9.5)	28 (38.4)^a^	54 (60.0)^a, b^	7 (58.3)^a^	< 0.001
Atrial fibrillation (%)	10 (47.6)	40 (54.8)	28 (31.1)^b^	7 (58.3)	0.014
Medications					
Diuretic (%)	10 (47.6)	48 (65.8)	55 (61.1)	9 (75)	0.366
ACEI/ARB/ARNI (%)	11 (52.4)	27 (37.0)	16 (17.8)^a, b^	3 (25)	0.003
β-Blocker (%)	11 (52.4)	45 (61.6)	37 (41.1)^b^	7 (58.3)	0.069
Spironolactone (%)	7 (33.3)	34 (46.6)	34 (37.8)	4 (33.3)	0.552
SGLT2i (%)	2 (9.5)	6 (8.2)	1 (1.1)	0 (0)	0.064
Cardiotonic (%)	0 (0)	6 (8.2)	6 (6.7)	4 (33.3)^a, b, c^	0.024
Iron agents (%)	0 (0)	11 (15.1)	12 (13.3)	3 (25)	0.128
Nutrition support (%)	4 (19.0)	19 (26.0)	38 (42.2)^a, b^	9 (75)^a, b, c^	0.002

	GNRI score				
	Normal (> 98, n = 57)	Low malnutrition risk (92 ≤ GNRI ≤ 98, n = 38)	Medium malnutrition risk (82 ≤ GNRI < 92, n = 78)	High malnutrition risk (< 82, n = 23)	
General situation					
Hospitalization days (days)	27 (16, 40)	28.5 (16.8, 39.3)	29 (18, 43.3)	42 (25, 56)^a, b, c^	0.037
Age (years)	92 (90, 94)	92.5 (90.5, 94)	93 (90, 94.25)	94 (91, 97)	0.149
Male (%)	53 (93.0)	35 (92.1)	75 (96.2)	19 (82.6)	0.175
Heart rate (beats/min)	73 (68, 79.5)	70 (63.5, 83.5)	73 (65, 79.25)	77 (70, 84)	0.559
Systolic blood pressure (mm Hg)	134 (119.5, 150)	138.5 (121, 150)	126 (120, 144)	135 (115, 140)	0.515
Diastolic blood pressure (mm Hg)	70 (63, 79)	70 (61.75, 77)	68 (61.75, 76)	70 (63, 79)	0.36
BMI (kg/m^2^)	25.9 (23.7, 28.1)	22.7 (21.3, 24.4)^a^	22 (20.6, 22.9)^a, b^	20.3 (19.0, 22.1)^a, b^	< 0.001
CONUT score	3 (2, 4)	4 (2, 5)	6 (4, 7)^a, b^	7 (6, 9)^a, b, c^	< 0.001
GNRI score	102.6 (99.6, 106.7)	94.5 (93.1, 96.3)^a^	87.8 (86.2, 89.8)^a, b^	78.7 (76.3, 80.5)^a, b, c^	< 0.001
Laboratory data					
Albumin (g/L)	37.63 ± 4.34	34.92 ± 0.66^a^	31.63 ± 2.49^a, b^	26.78 ± 2.44^a, b, c^	< 0.001
Lymphocyte (× 10^9^/L)	1.51 (1.10, 1.82)	1.40 (0.905, 1.835)	1.1 (0.735, 1.54)^a, b^	1.11 (0.66, 1.56)^a, b^	0.001
Cholesterol (mmol/L)	3.39 (2.70, 4.00)	3.75 (3.33, 4.49)	3.25 (2.91, 3.8525)	3.63 (3.16, 4.05)	0.067
Hemoglobin (g/L)	118 (99.5, 135.5)	117 (106.5, 126.25)	112 (100.75, 123.25)	99 (87, 109)^a, b, c^	0.004
C-reactive protein (mg/L)	2.6 (0.5, 14.25)	2.35 (0.5, 16.15)	12.1 (2, 44.325)^a, b^	18.2 (7.5, 55.1)^a, b^	< 0.001
Urea nitrogen (mmol/L)	10.2 (7.65, 13.9)	9.4 (7.2, 12.425)	8.9 (6.8, 13.375)	9.5 (7.2, 14)	0.454
Creatinine (µmol/L)	122.7 (81.4, 176.9)	96.9 (78.4, 136.4)	98.9 (76.7, 135.9)	102.9 (78.4, 125.8)	0.113
Prealbumin (mg/L)	221.79 ± 56.29	210.03 ± 51.45	184.5 ± 49.98^a, b^	149 ± 73.7^a, b^	< 0.001
Triglyceride (mmol/L)	1.02 (0.715, 1.32)	1.03 (0.675, 1.6325)	0.89 (0.66, 1.28)	0.79 (0.61, 1.21)	0.419
LDL-C (mmol/L)	1.63 (1.26, 2.28)	1.82 (1.21, 2.28)	1.68 (1.37, 2.17)	1.81 (1.42, 2.13)	0.916
HDL-C (mmol/L)	0.96 (0.79, 1.145)	1.15 (0.93, 1.47)^a^	0.97 (0.81, 1.14)^b^	1 (0.85, 1.33)	0.023
PCT (µg/L)	0.08 (0.04, 0.12)	0.05 (0.04, 0.11)	0.10 (0.05,0.21)^a, b^	0.16 (0.10, 0.39)^a, b, c^	< 0.001
IL-6 (ng/L)	10.65 (5.77, 20.14)	8.94 (5.62, 17.67)	17.74 (9.91, 37.84)^a, b^	25.6 (13.0, 52.23)^a, b^	< 0.001
NT-proBNP (pmol/L)	249 (208.5, 380.8)	278.5 (170.8, 373.4)	316.9 (209.9, 500.7)	361.4 (270.9, 724.4)^a, b^	0.012
TSAT (%)	0.27 (0.20, 0.34)	0.26 (0.23, 0.35)	0.27 (0.18, 0.35)	0.22 (0.14, 0.26)^a, b, c^	0.033
SF (µmol/L)	11.6 (8.4, 15.8)	12.4 (8.65, 13.525)	9.6 (6.75, 12.3)^a^	6.9 (5.4, 9)^a, b, c^	< 0.001
UIBC (µmol/L)	32.2 (25.95, 38.4)	30.35 (23.5, 37.93)	25.75 (20.18, 31.33)^a, b^	26.9 (22.4, 32.3)^a^	0.001
TIBC (µmol/L)	45.1 (37.2, 51.35)	40.45 (34.85, 51)	35.85 (30.28, 40.43)^a, b^	33.8 (28.2, 42.2)^a, b^	< 0.001
Echocardiographic data					
LVEF (%)	59 (56, 63.5)	61 (58, 64)	61 (57, 66.3)	60 (57, 66)	0.558
AOD (mm)	35 (42, 50)	35 (32, 37.25)	34 (31.75, 38)	34 (33, 36)	0.653
LAD (mm)	45 (42, 50)	43 (39.75, 45.5)^a^	41 (37, 44)^a^	39 (35, 41)^a, b^	< 0.001
LVDD (mm)	48 (45, 50)	49.5 (44.75, 53)	45 (43, 49)^a, b^	46 (42, 49)^a, b^	0.002
RVD (mm)	25 (23, 26)	24 (22.5, 26)	25 (22, 27)	23 (18, 26)	0.102
IVS (mm)	11 (10, 12)	10 (10, 11)^a^	11 (10, 12)^a^	11 (10, 12)	0.025
LVPW (mm)	11 (10, 12)	10 (10, 11)^a^	10 (9, 11)^a^	10 (10, 11)	0.020
Comorbidity					
Hypertension (%)	47 (82.5)	28 (73.7)	61 (78.2)	17 (73.9)	0.729
Diabetes (%)	27 (47.4)	16 (42.1)	32 (41.0)	7 (30.4)	0.579
Coronary heart disease (%)	55 (96.5)	37 (97.4)	75 (96.2)	20 (87.0)	0.264
COPD (%)	22 (38.6)	13 (34.2)	24 (30.8)	8 (34.8)	0.825
Chronic renal insufficiency (%)	32 (56.1)	12 (31.6)	33 (42.3)	10 (43.5)	0.119
Anemia (%)	24 (42.1)	13 (34.2)	37 (46.4)	17 (73.9)^a, b, c^	0.021
Atrial fibrillation (%)	34 (59.6)	14 (36.8)^a^	31 (39.7)^a^	6 (26.1)^a^	0.018
Medications					
Diuretic (%)	37 (64.9)	19 (50.0)	49 (62.8)	17 (73.9)	0.268
ACEI/ARB/ARNI (%)	26 (45.6)	9 (23.7)^a^	21 (26.9)	1 (4.3)^a, b, c^	0.002
β-Blocker (%)	35 (61.4)	17 (44.7)	35 (44.9)	13 (56.5)	0.211
Spironolactone (%)	27 (47.4)	10 (26.3)	32 (41.0)	10 (43.5)	0.223
SGLT2i (%)	3 (5.3)	4 (10.5)	2 (2.6)	0 (0)	0.217
Cardiotonic (%)	6 (10.5)	1 (2.6)	5 (6.4)	4 (17.4)	0.176
Iron agents (%)	5 (8.8)	6 (15.8)	10 (12.8)	5 (21.7)	0.447
Nutrition support (%)	10 (17.5)	11 (28.9)	31 (39.7)	18 (78.3)^a, b, c^	< 0.001

^a^P values < 0.05 vs. normal malnutrition or no risk of malnutrition. ^b^P values < 0.05 vs. mild malnutrition or low malnutrition risk. ^c^P values < 0.05 vs. moderate malnutrition or medium malnutrition risk. BMI: body mass index; CONUT: Controlling Nutritional Status; GNRI: Geriatric Nutritional Risk Index; LDL-C: low-density lipoprotein cholesterol; HDL-C: high-density lipoprotein cholesterol; PCT: procalcitonin; IL-6: interleukin-6; NT-proBNP: N-terminal pro-B-type natriuretic peptide; TSAT: transferrin saturation; SF: serum ferritin; UIBC: unsaturated iron-binding capacity; TIBC: total iron-binding capacity; LVEF: left ventricular ejection fraction; AOD: aortic diameter; LAD: left atrial diameter; LVDD: left ventricular diastolic diameter; RVD: right ventricular diameter; IVS: interventricular septum; LVPW: left ventricular posterior wall; COPD: chronic obstructive pulmonary disease; ACEI: angiotensin-converting-enzyme inhibitor; ARB: angiotensin II receptor blocker; ARNI: angiotensin-receptor-neprilysin inhibitor; SGLT2i: sodium-dependent glucose transporters 2 inhibitor.

### Prognosis of different groups of patients under CONUT score and GNRI score

Over a median follow-up of 11.5 months, 83 (42.3%) patients died. As the CONUT scores increased, the rates of all-cause death, cardiovascular death, all-cause readmission, and HF-related readmission rose significantly (P < 0.05). Patients in the higher nutritional risk group based on the GNRI scores had notably higher rates of all-cause death, all-cause readmission, and HF-related readmission (P < 0.05), whereas no significant inter-group difference was observed for cardiovascular death (P = 0.136), as shown in Table 2.

**Table 2:** Prognosis Outcomes of Patients Stratified by CONUT and GNRI Score

Prognosis	CONUT score				P value
	Normal (0–1, n = 21)	Mild malnutrition (2–4, n = 73)	Moderate malnutrition (5–8, n = 90)	Severe malnutrition (9–12, n = 12)	
All-cause death (%)	1 (4.8)	13 (17.8)	57 (63.3)^a, b^	12 (100)^a, b^	< 0.001
Cardiovascular death (%)	0 (0.0)	4 (5.5)	14 (15.6)	3 (25.0)	0.019
All-cause readmission (%)	11 (52.4)	59 (80.8)^a^	84 (93.3)^a, b^	10 (83.3)^a^	< 0.001
HF-related readmission (%)	2 (9.5)	23 (31.5)^a^	55 (61.1)^a, b^	10 (83.3)^a, b^	< 0.001

Prognosis	GNRI score				P value
	Normal (> 98, n = 57)	Low malnutrition risk (92 ≤ GNRI ≤ 98, n = 38)	Medium malnutrition risk (82 ≤ GNRI < 92, n = 78)	High malnutrition risk (< 82, n = 23)	
All-cause death (%)	11 (19.3)	9 (23.7)	41 (52.6)^a, b^	22 (95.7)^a, b, c^	< 0.001
Cardiovascular death (%)	2 (3.5)	4 (10.5)	12 (15.4)	3 (13.0)	0.136
All-cause readmission (%)	43 (75.4)	29 (76.3)	71 (91.0)^a^	21 (91.3)	0.038
HF-related readmission (%)	15 (26.3)	12 (31.6)	46 (59.0)^a, b^	17 (73.9)^a, b^	< 0.001

^a^P values < 0.05 vs. normal malnutrition or no risk of malnutrition. ^b^P values < 0.05 vs. mild malnutrition or low malnutrition risk. ^c^P values < 0.05 vs. moderate malnutrition or medium malnutrition risk. CONUT: Controlling Nutritional Status; GNRI: Geriatric Nutritional Risk Index.

### Survival analysis of different groups of patients under CONUT score and GNRI score

For CONUT scores, higher scores were linked to increased all-cause mortality risk and decreased survival probability, and for GNRI scores, lower scores were associated with increased all-cause mortality risk and decreased survival probability, as shown in Figure 2.

**Figure 2:**
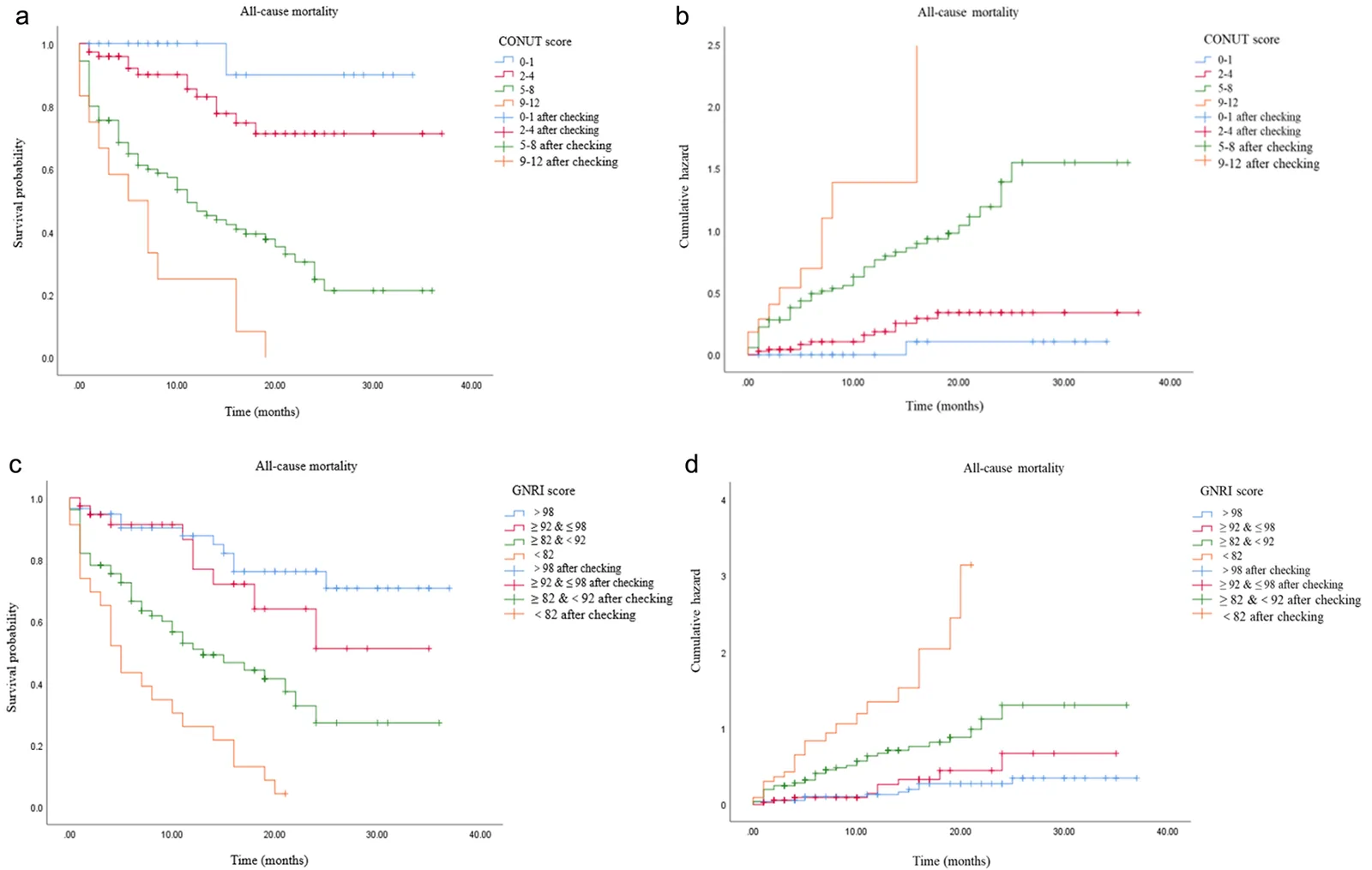
Kaplan–Meier survival curves. (a) Survival probability stratified by CONUT score. (b) Cumulative hazard stratified by CONUT score. (c) Survival probability stratified by GNRI score. (d) Cumulative hazard stratified by GNRI score. CONUT: Controlling Nutritional Status; GNRI: Geriatric Nutritional Risk Index.

Multivariable Cox proportional-hazards regression was performed with all-cause mortality as the dependent variable, adjusting for age, hypertension, diabetes, chronic renal insufficiency, GNRI score, CONUT score, and NT-proBNP. The analysis revealed that CONUT score and NT-proBNP were independently associated with higher all-cause mortality. By contrast, GNRI score was independently associated with lower mortality, indicating that better nutritional status correlated with reduced death risk. No significant associations were observed for age, hypertension, diabetes, or renal insufficiency (all P > 0.05), as shown in Table 3.

**Table 3:** Multivariable Cox Proportional Hazards Regression for All-Cause Mortality in Elderly Patients With HFpEF

	P value	HR (95% CI)
Age	0.318	1.208 (0.974–1.085)
Hypertension	0.130	0.671 (0.400–1.125)
Diabetes	0.124	1.444 (0.904–2.307)
Chronic renal insufficiency	0.283	1.288 (0.812–2.042)
GNRI score	0.043	0.962 (0.927–0.999)
CONUT score	0.001	1.366 (1.181–1.579)
NT-proBNP	0.017	1.000 (1.000–1.001)

CI: confidence interval; CONUT: Controlling Nutritional Status; GNRI: Geriatric Nutritional Risk Index; HR: hazard ratio; NT-proBNP: N-terminal pro-B-type natriuretic peptide.

### Consistency and difference between CONUT score and GNRI score in evaluating nutritional status of elderly patients with HFpEF

The two scores showed poor consistency in assessing nutritional status of elderly HFpEF patients (P < 0.01, κ = 0.235). Both scores showed predictive capacity for all-cause mortality. The AUC was 0.86 (95% CI 0.80–0.91) for CONUT (optimal cut-off: 5.5, maximum Youden’s index: 0.593), 0.79 (95% CI 0.73–0.86) for GNRI (optimal cut-off: 83.83, maximum Youden’s index: 0.498), and 0.87 (95% CI 0.82–0.92) for the combined score. The predictive performance of CONUT alone and the combined score was significantly better than that of GNRI alone (P < 0.01), whereas no statistically significant difference was observed between CONUT alone and the combined score (P = 0.078), as shown in Figure 3.

**Figure 3:**
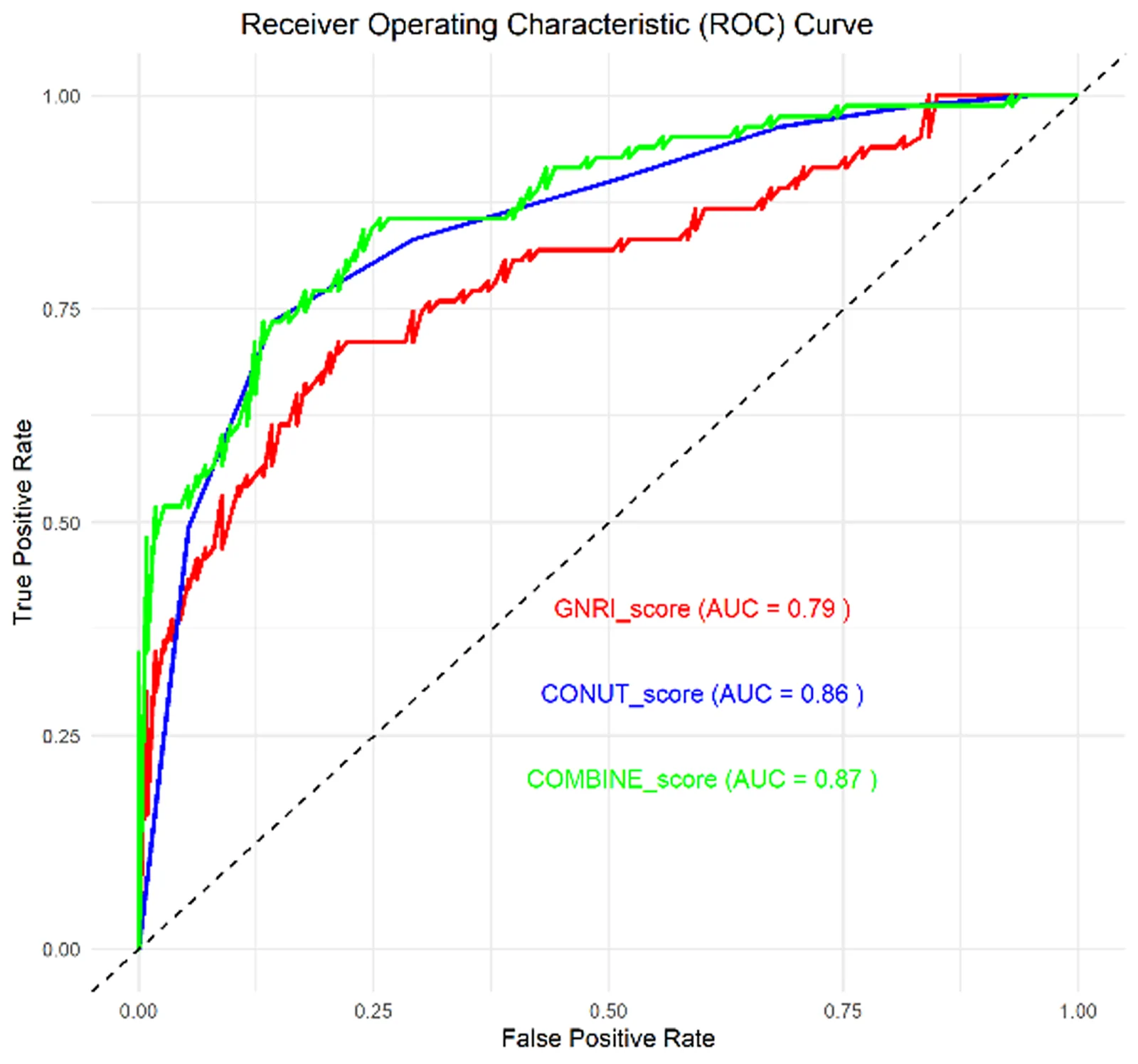
Receiver operating characteristic (ROC) curve of CONUT score, GNRI score, and the combined scores. AUC: area under the curve; CONUT: Controlling Nutritional Status; GNRI: Geriatric Nutritional Risk Index.

Similarly, NRI analysis showed that compared with the GNRI score, the NRI of the CONUT score was 0.580 (95% CI: 0.309–0.852, P = 0.001), and the NRI of the combined score was 0.760 (95% CI: 0.499–1.022, P = 0.001), both reaching statistical significance. By contrast, the NRI was 0.083 (95% CI: −0.197 to 0.363, P = 0.560) for the combined score versus the CONUT score, without statistical significance.

## Discussion

HFpEF is the most prevalent subtype of HF in the elderly population and a major contributor to mortality among hospitalized elderly patients [2]. Malnutrition is common in this population, driven by multifactorial mechanisms including systemic metabolic alterations, diminished gastrointestinal function, and underlying comorbidities [12]. Accumulating evidence suggests that malnutrition is associated with adverse outcomes in HF patients, potentially through its effects on myocardial energy metabolism and immune function [9, 10, 13]. However, previous studies have mostly focused on the overall HF population or HFrEF, whereas the association between nutritional status and prognosis in elderly patients with HFpEF remains relatively under-explored.

Currently, various clinical tools are available for nutritional risk screening [14, 15]. The Nutritional Risk Screening 2002 (NRS 2002) is widely used for inpatients but significantly influenced by the reliability of patient medical history. Subjective Global Assessment (SGA) and Mini Nutritional Assessment (MNA) require professional medical intervention and involve subjective questions, making it challenging to obtain accurate data from patients with unclear expressions, particularly the elderly [16, 17]. In contrast, CONUT and GNRI are objective, laboratory-based scoring systems that are less affected by subjective factors. The CONUT score integrates serum ALB, TC, and LY to reflect protein reserves, caloric status, and immune function [4], while the GNRI is derived from serum ALB and BMI [5]. Both tools have been validated as prognostic predictors in various clinical settings, including cardiovascular disease [6–8, 18].

Regarding the pathophysiological links between malnutrition and HF, several mechanisms have been proposed in the literature. Fluid retention may contribute to intestinal edema and anorexia, affecting nutrient intake and absorption [3]. Chronic inflammation and neurohormonal activation may promote protein and adipose tissue catabolism, leading to weight loss and cachexia [19–21]. In our study, patients with poorer nutritional status had higher levels of inflammatory markers and lower lipid metabolism indices, which aligns with these previously described associations. However, as this study is purely observational and did not assess pathways such as intestinal barrier function, inflammatory cytokine signaling, or neurohormonal activation, the available data cannot establish mechanistic causality between the two.

Previous studies have demonstrated that the CONUT and GNRI scores were associated with readmission and all-cause mortality in patients with HF [22, 23]. A meta-analysis has also indicated that malnutrition, defined by a CONUT score ≥ 2, is associated with an elevated risk of all-cause mortality, with HF patients experiencing a 1.92-fold increased risk of death from any cause [24]. However, these studies neither stratified analyses by age (i.e., focusing on the elderly) nor distinguished HF subtypes. This study investigated the association between the two scores and prognosis in elderly patients with HFpEF. The results revealed that the CONUT score was an independent risk factor for all-cause mortality in elderly patients with HFpEF; each one-point increase in the CONUT score was associated with a 36.6% higher risk of all-cause mortality. The GNRI score was an independent protective factor. Higher GNRI scores, reflecting better nutritional status, were associated with lower mortality risk, which is consistent with previous findings in the overall HF population [25].

Notably, we observed that the high-nutritional-risk group (defined by GNRI) had smaller left atrial diameter (LAD) and left ventricular diastolic diameter (LVDD), and that interventricular septum (IVS) and left ventricular posterior wall (LVPW) differed across GNRI categories. One possible explanation is that patients with poor nutritional status more often exhibit a non-obese HFpEF phenotype, with smaller body surface area and lower preload, leading to smaller absolute cardiac chamber dimensions. This observation may reflect the known heterogeneity of HFpEF, but our cross-sectional data cannot determine whether nutritional status is directly associated with cardiac remodeling. These findings are hypothesis-generating and require confirmation in dedicated mechanistic or longitudinal studies.

Poor consistency was observed between the CONUT and GNRI scores, indicating that the two nutritional-assessment tools focus on different dimensions and cannot substitute for one another in clinical practice. ROC and NRI analyses in the present study demonstrated that the combined-score model showed significantly better predictive performance for all-cause mortality among elderly patients with HFpEF compared with the GNRI score; however, no obvious incremental benefit was observed relative to CONUT alone. These findings suggest that the prognostic value of the combined model is mainly derived from the CONUT score. We speculate that this result may be attributed to water-sodium retention in HF patients, which disturbs BMI and further leads to the weaker predictive performance of GNRI.

As the aging population increases, greater attention should be paid to elderly patients with HFpEF complicated by malnutrition. Current HF guidelines [2] recommend that all HF patients undergo nutritional risk assessment. Future prospective, multicenter studies with larger sample sizes and comprehensive multivariable adjustment are warranted to validate the prognostic value of CONUT and GNRI in elderly HFpEF patients. The dynamic changes in nutritional status and the potential impact of nutritional interventions on clinical outcomes should also be explored. Additionally, given the heterogeneity of HFpEF, nutritional indicators may aid in subtype identification, although their clinical utility in this regard needs to be confirmed by dedicated studies.

### Limitations

This study was a single-center retrospective analysis with a relatively small sample size, especially for subgroup analyses. Given that most of the consecutively enrolled participants were male, selection bias may exist. Nutritional status was assessed solely based on admission laboratory data; therefore, the prognostic associations of temporal changes in nutritional status and nutritional intervention could not be evaluated. This study only confirmed that there was no significant difference in the prevalence of coronary heart disease among groups. Given the lack of comparison of lipid-lowering medication use, the confounding effect of lipid-lowering drugs cannot be fully excluded. We used standard ROC based on binary endpoint rather than time-dependent ROC for survival outcomes. Further large-sample prospective studies are needed to validate our findings.

### Conclusions

Malnutrition is a prevalent complication among elderly patients with HFpEF, significantly associated with prolonged hospital stays, all-cause death, and higher rates of HF-related readmission. The CONUT and GNRI scores are widely used in clinical nutritional assessment and cannot fully substitute for one another. Compared with the GNRI score alone, combined evaluation of the two scores showed improved predictive performance for adverse prognosis in elderly patients with HFpEF, whereas no incremental predictive value was observed relative to the CONUT score alone.

## Data Availability

The datasets used and analyzed during the current study are available from the corresponding author on reasonable request.

## Abbreviations

ACEI: angiotensin-converting-enzyme inhibitor
AOD: aortic diameter
ARB: angiotensin II receptor blocker
ARNI: angiotensin-receptor-neprilysin inhibitor
AUC: area under the curve
BMI: body mass index
CONUT: Controlling Nutritional Status
COPD: chronic obstructive pulmonary disease
GNRI: Geriatric Nutritional Risk Index
HDL-C: high-density lipoprotein cholesterol
HFpEF: heart failure with preserved ejection fraction
IL-6: interleukin-6
IVS: interventricular septum
LAD: left atrial diameter
LDL-C: low-density lipoprotein cholesterol
LVDD: left ventricular diastolic diameter
LVEF: left ventricular ejection fraction
LVPW: left ventricular posterior wall
NT-proBNP: N-terminal pro-B-type natriuretic peptide
PCT: procalcitonin
ROC: receiver operating characteristic
RVD: right ventricular diameter
SF: serum ferritin
SGLT2i: sodium-glucose cotransporter 2 inhibitor
TIBC: total iron-binding capacity
TSAT: transferrin saturation
UIBC: unsaturated iron-binding capacity
